# Analysis of novel geometry-independent method for dialysis access pressure-flow monitoring

**DOI:** 10.1186/1742-4682-5-22

**Published:** 2008-11-05

**Authors:** William F Weitzel, Casey L Cotant, Zhijie Wen, Rohan Biswas, Prashant Patel, Harsha Panduranga, Yogesh B Gianchandani, Jonathan M Rubin

**Affiliations:** 1 School of Medicine, University of Michigan, Ann Arbor, MI, USA; 2 College of Engineering, University of Michigan, Ann Arbor, MI, USA

## Abstract

**Background:**

End-stage renal disease (ESRD) confers a large health-care burden for the United States, and the morbidity associated with vascular access failure has stimulated research into detection of vascular access stenosis and low flow prior to thrombosis. We present data investigating the possibility of using differential pressure (ΔP) monitoring to estimate access flow (Q) for dialysis access monitoring, with the goal of utilizing micro-electro-mechanical systems (MEMS) pressure sensors integrated within the shaft of dialysis needles.

**Methods:**

A model of the arteriovenous graft fluid circuit was used to study the relationship between Q and the ΔP between two dialysis needles placed 2.5–20.0 cm apart. Tubing was varied to simulate grafts with inner diameters of 4.76–7.95 mm. Data were compared with values from two steady-flow models. These results, and those from computational fluid dynamics (CFD) modeling of ΔP as a function of needle position, were used to devise and test a method of estimating Q using ΔP and variable dialysis pump speeds (variable flow) that diminishes dependence on geometric factors and fluid characteristics.

**Results:**

In the fluid circuit model, ΔP increased with increasing volume flow rate and with increasing needle-separation distance. A nonlinear model closely predicts this ΔP-Q relationship (R^2 ^> 0.98) for all graft diameters and needle-separation distances tested. CFD modeling suggested turbulent needle effects are greatest within 1 cm of the needle tip. Utilizing linear, quadratic and combined variable flow algorithms, dialysis access flow was estimated using geometry-independent models and an experimental dialysis system with the pressure sensors separated from the dialysis needle tip by distances ranging from 1 to 5 cm. Real-time ΔP waveform data were also observed during the mock dialysis treatment, which may be useful in detecting low or reversed flow within the access.

**Conclusion:**

With further experimentation and needle design, this geometry-independent approach may prove to be a useful access flow monitoring method.

## Background

Dialysis access blood volume flow and pressure may be helpful parameters in end-stage renal disease (ESRD) vascular access monitoring. [1-5] The magnitude of the clinical problem is well recognized, with 330,000 dialysis patients with ESRD in the U.S., and the cost of maintaining dialysis access in the care of these patients is over $1 billion in the U.S. alone, which represents approximately 10% of the total cost of dialysis care.[6,7] The recently updated National Kidney Foundation (NKF) Dialysis Outcomes and Quality Initiative (DOQI) recommendations have reaffirmed the recommendation for monitoring using monthly measurement of flow or static venous pressure as the preferred methods.[8] Monthly flow monitoring may lead to as much as a 50% reduction in access failure,[9] yet this number still represents 25% of patients with grafts experiencing failure (thrombosis or clotting) per year, which requires emergency treatment to re-establish flow. Divergent opinions exist about the utility of flow monitoring, partly fueled by the relatively infrequent (e.g., monthly) flow monitoring interval. [10-12] Since it may be practical to follow access pressure more frequently,[13] some have advocated pressure monitoring over flow monitoring.[14] Additionally, it should be noted that other data support the cost effectiveness of access flow monitoring even when performed less frequently,[15] and that the combined sensitivity and specificity improves,[16] and cost effectiveness improves,[17] when flow monitoring frequency is increased.

Our group is investigating the possibility of using differential pressure (ΔP) monitoring to estimate access flow for dialysis access monitoring, with the current study aimed at developing and testing an access geometry-independent algorithm that is convenient to perform throughout dialysis or at least at every dialysis session. The underlying assumption is that flow along with pressure monitoring may be a more complete representation of the hemodynamic status of the access. Furthermore, frequent and convenient flow estimations may improve monitoring by determining each patient's mean access flow and standard deviation in flow. Additionally, this would allow the change in access blood flow with ultrafiltration and blood pressure reduction to be followed, just as blood pressure and various machine parameters are followed during dialysis. However, several engineering problems must be addressed to make this approach clinically practical.

While pressure measurements within the access have been used as an indicator of stenosis (which partially obstructs flow and alters access pressure), pressure differences within the dialysis graft or fistula have not typically been used to estimate flow. This is primarily because well-established fluid dynamics models require knowledge or estimation of access geometry, needle separation, and fluid properties, such as viscosity, to determine flow.[18] This study derived experimental data on the relationship between access flow and ΔP between two dialysis access needles in a model of the arteriovenous graft (AVG) vascular circuit. This geometry-dependent data was used to devise methods and perform experiments that estimate access flow using ΔP and variable dialysis pump speeds while being mathematically independent of geometric factors and fluid characteristics. We present a potentially useful geometry-independent method, modeling data, and experimental results for flow determination using intra-access ΔP and its dependence on dialysis pump speed. Implementation of this method will require the development of new dialysis needle technology or intra-access ΔP measurement devices to allow for intra-access pressure measurement during dialysis, work that is currently in progress. These data suggest that this approach or subsequent permutations may result in easy to use, operator-independent alternative methods of access monitoring to improve future access monitoring strategies.

## Materials and methods

### Experimental Steady-Flow AVG Circuit

A fluid circuit model of the AVG vascular circuit was developed to study the relationship between access flow (Q) and the ΔP between two dialysis access needles placed 2.5, 5, 10, 15, and 20 cm from one another within the circuit. A Masterflex Console Drive non-pulsatile blood roller pump (Cole Parmer, Vernon Hills, IL) was utilized to draw a glycerol-based fluid, with a kinematic velocity of 0.029 cm^2^/s (corresponding to a hematocrit of approximately 37%), from a fluid reservoir. The fluid was channeled to a Gilmont flow meter (Thermo Fisher Scientific, Waltham, MA), which was calibrated using the 37% glycerol solution. The fluid subsequently flowed back to the fluid reservoir before returning to the pump in a closed circuit. The polyvinyl tubing used in the circuit had inner diameters of 4.76 mm (3/16"), 6.35 mm (1/4"), and 7.95 mm. The 16-guage needles were primed with the 37% glycerol solution, and a digital pressure monitor (model PS409, Validyne, Northridge, CA) was used to directly measure ΔP between the "upstream" and "downstream" needles, in millimeters of mercury. Digital data were downloaded to a PC using data acquisition hardware and software (DATAQ Instruments, Akron, OH). During steady-state flow, the pressure monitor was observed for 20–30 seconds, until the reading stabilized, before recording the value.

Experimental values were compared to the theoretical results from two well-established steady flow models, which are first-order approximations to pulsatile flow. One of the best described solutions for laminar flow through a straight circular tube of constant cross section is the Hagen-Poiseuille (hereafter, Poiseuille) equation.[19] This equation for laminar flow was evaluated as follows:[18]

(1) $$\Delta \text{P}=\frac{128\mu {\text{QL}}_{\text{G}}}{\pi {\text{D}}_{\text{G}}^{4}},$$

in which μ is the dynamic viscosity of the liquid, L_G _is the length of the graft, and D_G_^4 ^refers to the inner diameter of the graft raised to the 4th power. With this equation, the relationship between ΔP and Q is linear. For each tube inner diameter and at each distance of separation, ten measurements were taken at each flow rate. The mean, standard deviation, and correlation coefficient values between Poiseuille's model and the experimental data were calculated.

Similarly, Young's general expression for a flow rate-dependent pressure drop between two locations where a liquid flows through a channel was evaluated:[20,21]

(2) ΔP = R_a_V + R_b_V^2^,

where ΔP represents the pressure difference between the downstream and upstream locations, V is area-averaged flow velocity in an unobstructed vessel, and R_a _and R_b _are coefficients that depend on obstacle geometry and fluid properties. Young's expression was chosen as one of the simplest models incorporating higher order terms (Q raised to the second power) that may be used to characterize turbulent flow resulting from higher velocity flow conditions with higher Reynolds numbers, geometry-induced flow disturbances from vessel diameter change or intraluminal irregularities, as well as cannulas within the flow path. [18-20]

Correlation coefficients were calculated to evaluate the fit of the data to Poiseuille's linear model and Young's second-order polynomial equation. To establish dynamic similitude between our *in vitro *model and the *in vivo *AVG circuit, Reynolds numbers were calculated for each flow rate and for each of the three separate AVG inner diameters based on the expression Re = ρvD/μ, where ρ is the density of the fluid (1090.04 kg/m^3^), v is the velocity 4 Q/πD^2^, D is the inner diameter of the tube, and μ is the dynamic viscosity (0.0032 kg/ms).[18]

### Experimental Variable Flow Dialysis Circuit

**Figure 1:**
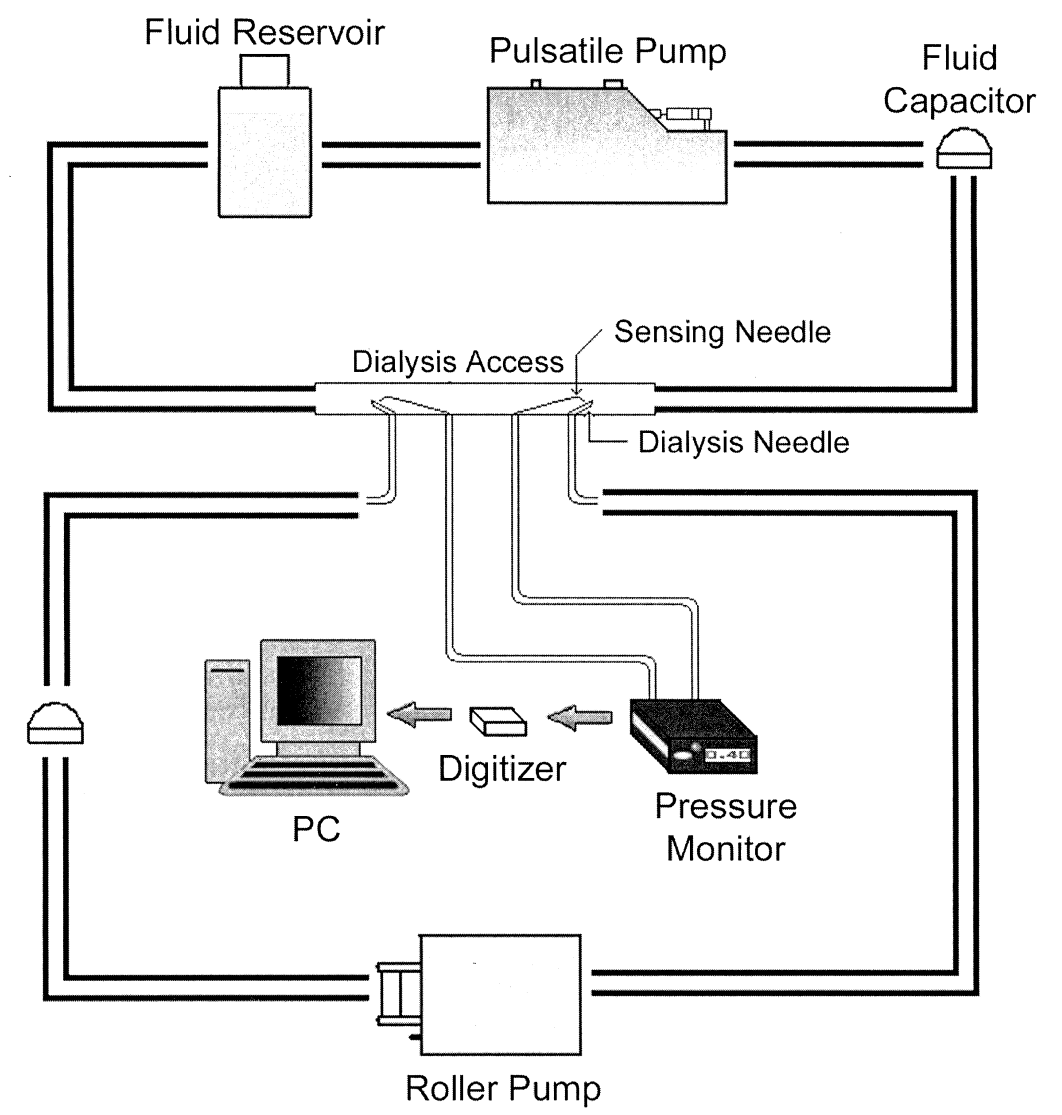
**Schematic of flow circuit**. Model of patient blood flow system to test geometry-independent algorithms for flow determination.

To test the geometry-independent algorithms for flow determination, we constructed a laboratory flow phantom system comprising the dialysis blood pump system described above communicating in parallel with a patient blood circuit. Access diameters of 4.76- and 6.35-mm inner diameter were used to approximate AVG inner diameters. The dialysis circuit was assembled to generate measurable flow rates using the adjustable non-pulsatile roller pump, the Gilmont flow meter calibrated to ensure the accuracy of simulated dialysis pump speeds ranging from 0 to 500 mL/min, and an S-110 digital flow meter (McMillan, Georgetown, TX). The dialysis circuit was connected to the dialysis graft with 15-gauge dialysis needles (Sysloc, JMS Singapore PTE LTD, Singapore). The dialysis access was simulated using vinyl tubing (Watts Water Technologies, North Andover, MA). The patient blood circuit was modeled using a pulsatile adjustable blood pump (Harvard Apparatus, Holliston, MA) in series with a bubble trap (ATS Laboratories, Bridgeport, CT) to act as a large capacitance vessel. This was in series with the access graft, which had been cannulated with the dialysis needles from the dialysis circuit. A downstream air trap was also located within the patient circuit. Pressure sensing within the conduit was achieved using 21-gauge spinal needles positioned with needle tips 5, 2 and 1 cm from the upstream-facing arterial needle and the downstream-facing venous needle tip. The model flow circuit is depicted in Figure 1.

Experimental data were collected at pulsatile pump speeds of 400, 800, and 1200 mL/min, simulating these dialysis access flow rates, and the dialysis pump speed was varied from 0 to 400 mL/min, simulating dialysis pump "off" and "on" conditions, respectively, for each access diameter (4.76 and 6.35 mm), with 20-cm dialysis needle separation, at variable pressure sensor needle distances (1 to 5 cm) from the intraluminal dialysis needle tip. Fluid viscosity was 0.29 centistokes, corresponding to hematocrit of 37%.

### Derivation of Geometry-independent Models

The pressure drop between needles may be represented by numerous fluid dynamics models representing the blood flow through a dialysis conduit. The pressure in these models depends to varying degrees on polynomial expressions of the flow raised to integer or fractional powers.[18,20] Although many of these are straightforward algebraic expressions, the models become rather complicated to implement in clinical practice because, in addition to relating flow and pressure, they contain additional parameters such as the dialysis needle separation (or distance along the dialysis access where pressure difference is measured), access diameter (or potentially more complicated forms expressing dialysis access geometry), and factors affecting fluid flow such as blood viscosity. With any of these relationships, it is understood that pressure is always with respect to a reference pressure. Therefore, if needle pressure is used, the pressure difference between the arterial (PA) and venous (PV) needle sites in the dialysis access is the ΔP between sensors (ΔPAV). Since PV, as it is used in dialysis access monitoring currently, is the relative pressure between the venous needle site and atmospheric pressure, and since PA is the relative pressure between the arterial needle site and atmospheric pressure, PV-PA gives the relative pressure between the two needle sites indirectly using two pressure readings with the same reference pressure (in this case atmospheric pressure), and ΔPAV may be determined by direct measurement of the pressure difference between the two points using a single pressure measurement transducer.

In general, any mathematical relationship (so-called function F) that allows one to map (in a mathematical sense) the two or more pressure measurements to determine the volume flow (Q) or velocity (v) in the blood circuit may be used. This may take the general form:

(3) F(PV, PA) = Q

Alternatively, their inverse relationships may be utilized. These functions may be determined from theoretical principles, or F (or approximations of F) may be determined from values derived from experiments or clinical data and applied to make measurements of Q or v in practice.

A pulsatile-flow model relating pressure to flow is not used here; rather, we employ a first-order approximation with steady flow to allow us to test the method of measurement being evaluated. Based on theoretical grounds of using laminar flow with linear pressure-flow relationships and our experimental system showing pressure-flow relationships fitting a second-order polynomial, we selected two relationships to test, one in which pressure is related to the square of flow and one in which pressure is related linearly to flow. Other mathematical relationships may take alternative algebraic, numerical, or other mathematical forms.

### Using Diverted Dialysis Pump Flow To Determine Access Flow

Methods that exploit the decreasing blood flow between the needles within the access as blood is pumped through the circuit during dialysis take advantage of changes in pressure within this segment of the access. The effects of needle tip flow must be considered whenever the needle tip flow disturbance is near the pressure transducer; precisely how near or far the transducer must be from the needle tip must be determined from modeling, such as computational fluid dynamics (CFD), and experimental results, such as those presented in this study.

One physical system exploiting this method involves pressure transducers integrated on the outside of the shaft. The measurement method outlined below will be tested with needle designs in the future based on the experimental results presented in this study. A micro-electro-mechanical systems (MEMS) manufacturing method referred to as micro-electro-discharge machining (EDM) has been used for three-dimensional machining of cavities in needle shafts for MEMS sensor integration within needles.[22] The possibility of using this type of approach is also supported by our previous work using analogous extracorporeal measurement methods employing Doppler signals.[16,23,24]

Geometry- and fluid-dependent models can be used with any ΔP monitoring system.[20] However, given the uncertainty in the physical system and changes in vessel geometry that may occur over time, it may be advantageous to use geometry-independent modeling as a means of independently validating the measurements. In general, geometry-independent modeling can be performed if a tractable modeling relationship can be developed, exploiting the flow-dependent differential changes within the access, between the needles, as a result of changing the dialysis pump speed. The access blood flow rate (QA) depends on numerous factors, including systemic blood pressure and central venous pressure (reflecting pre- and post-access pressure gradients), access geometry (and thereby resistance), and blood viscosity, to name a few. Two needles are introduced into the access lumen during conventional dialysis; one for the removal of blood (arterial) to pass through the dialysis circuit and one for the return of blood (venous) to the circulation. For the purposes of testing this ΔP-based method, the arterial needle is facing upstream and the venous needle is facing downstream. The flow through the graft or fistula remaining downstream (QR) from the arterial needle will decrease during dialysis as a function of the blood flowing through the dialysis circuit at a blood pump flow rate (QB). To the extent that the net flow through the system does not change, this flow rate through the portion of the access between the dialysis needles (QR) will follow the relationship QR = QA - QB.[23,24] Other modeling functions can be constructed to model net changes in QA as a function of QB, but are not considered here for the sake of simplicity.

The ΔP between the needles will decrease as QB increases and QR decreases. While other observable signals that are predictably related to volume flow may have utility in this method, we will focus on ΔP (the pressure difference between the needles). The signal ΔP is measured and related mathematically to QB using a modeling function constructed for this signal F(QB) based on the measured values such that ΔP = F(QB). This modeling function may take the form of any algebraic or numerical function (preferably, but not necessarily, one-to-one in the range and domain of interest): linear, polynomial, exponential or otherwise. As QR decreases with increasing QB, the signal ΔP = F(QB) will decrease. As QR approaches zero, ΔP will approach zero, or a known value for ΔP that corresponds to zero blood flow QR. For our purposes in evaluating this method, zero or near zero time-averaged mean ΔP will correspond to zero volume flow QR. We can define this value using the modeling function as the signal S0 = F(0). This value for F(0) corresponds to the value for QB = QA, since QR = 0. QB at the value QA can be solved by calculating the projected intercept of the modeling function where ΔP = 0 or the known value for ΔP corresponding to zero mean flow between the needles. These calculations can be performed numerically by determining the inverse function of the modeling function or by solving them algebraically. To evaluate the method most simply, we evaluated a quadratic and linear form of the relationship between ΔP and access flow Q, with two dialysis pump speeds (pump "on" and pump "off"). For one expression, we have ΔP = CQ, in general, where C is a parametric constant containing geometric and rheologic factors. We define P_off _= CQA and P_on _= C(QA - QB) as the ΔP for pump off and pump on, respectively. Solving for the access flow QA gives the *linear model*:

(4) QA = QB/(1 - P_on_/P_off_).

For a second expression, we have ΔP = C(QA)^2^, and we define P_off _= C(QA)^2 ^and P_on _= C(QA - QB)^2 ^as the ΔP for pump off and pump on, respectively. Solving for the access flow QA gives the *quadratic model*:

(5) QA = QB/(1 - √(P_on_/P_off_)),

where QA depends on QB and the square root of the ratio of P_on _and P_off_. Importantly, notice that all of the geometric access and needle position parameters as well as the blood viscosity parameters contained in the term C have been eliminated from Equations 4 and 5. Therefore, although these parameters may be helpful in estimating flow from pressure, we have developed a method and derived an expression for determining flow from pressure that does not depend on these factors.

### Real-time Flow Estimation

An expression for real-time flow estimation (without altering the pump rate) can be tested using these experimental data. A parametric value for C (geometric and rheologic factors) can be used for C and estimated from the variable flow method: C = P_off_/(QA)^2^. Substituted into P_on _= C(QA - QB) and solving for QA gives

(6) QA = QB + √(P_on_/C),

where QA can be followed in real time without altering the pump rate by tracking the square root of the ratio of ΔP with pump on (P_on_) and C and adding this to the pump rate QB.

An analogous relationship can be determined using Equation 4, yielding

(7) QA = QB + P_on_/C,

should pressure vary linearly with flow. It should be noted that in practice it is anticipated that the pump may be briefly paused to re-calculate C to adjust for factors that may change during dialysis (e.g., ultrafiltration raising the hematocrit and altering viscosity) and then restarted to resume tracking QA in real time. Similarly, because experimental data and CFD results demonstrate a combination of linear (laminar) and quadratic (turbulent) flow patterns, we would anticipate that a geometry-independent model may represent a combination of these models. Most simply this may be an average of Equations 4 and 5 to yield the *combined model*:

(8) QA = (QB/2)(1/(1 - P_on_/P_off_) + 1/(1 - √(P_on_/P_off_)),

or a more complex combination with components accounting for laminar and turbulent flow patterns. The important feature of any of these models is that they are geometry and viscosity independent. We note that in the above, all flows are considered as time-averaged means to eliminate the need for phase information.

## Results

### Geometry-dependent Modeling

For each of the three tubes of varying inner diameter, ΔP increases as the volume flow rate increases, and there is a consistent increase in measured ΔP with increasing needle-separation distance. The non-linear curves demonstrate an apparent polynomial ΔP dependence on flow rate. This relationship appears to be more pronounced at needle separations >2.5 cm.

The data for each of the three tubes of varying inner diameter were matched to Poiseuille's (laminar flow) and Young's (turbulent flow) equations for Reynolds numbers less than and greater than, respectively, an approximate transitional value of 2100, where the transition between laminar and turbulent flow usually occurs.[25] For all tube diameters and needle separation distances, correlation coefficients were consistently higher (R^2 ^> 0.9828) for Young's equation compared with Poiseuille's (0.8449–0.9484). For the 4.76-mm tube, Reynolds numbers were <2100 for all flows <1387 mL/min. For the 6.35-mm tube, only the 1968-mL/min flow demonstrated a Reynolds number >2100. All Reynolds numbers were <2100 for the 7.95-mm-inner-diameter tube.

As graft inner diameter decreases, the mean ΔP also predictably increases. In addition, as Q increases for a given inner diameter, mean ΔP increases, with this relationship being most pronounced for the 4.76-mm-diameter tube. One final observation from the steady flow experiments is that ΔP increases with increasing distance between the two access needles. This relationship becomes more pronounced as the access flow increases, with the magnitude of the mean ΔP values being substantially greater using the 4.76-mm vs. the 7.95-mm-inner-diameter tube.

### Computational Fluid Dynamics (CFD) Modeling

**Figure 2:**
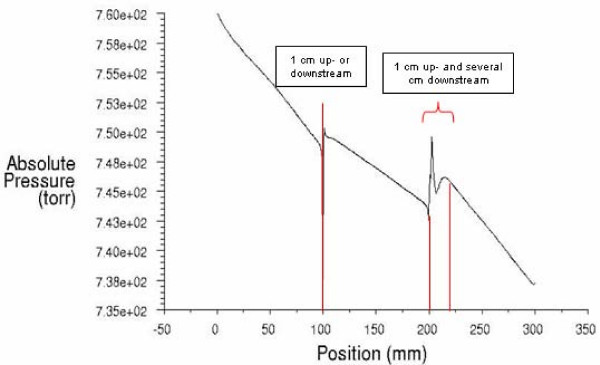
**Pressure as function of needle position**. Absolute pressure vs. position of arterial and venous needles within access with flow 1200 mL/min, pump on at 400 mL/min.

A family of CFD modeling curves was generated using FLUENT software (version 6.3, Fluent, Inc, Lebanon, NH). The pressure at the entrance of the tubing was set at atmospheric pressure (760 mmHg). The main meshing element applied to the cylinder geometry was "Tet/Hybrid," which specifies that the mesh is composed primarily of tetrahedral elements but may include hexahedral, pyramidal, and wedge elements where appropriate. In this model a "sink" is introduced upstream within the dialysis access to model the blood being drawn from the dialysis access through the arterial needle to the dialysis machine at a pump rate of 400 mL/min. A "source" is introduced downstream at a needle separation distance of 10 cm to model the venous needle returning blood to the dialysis access at a flow rate of 400 mL/min. ΔP is plotted along the y-axis, with distance along the vascular access plotted along the x-axis, thereby plotting the pressure drop along the length of the access longitudinally for a family of access flows Q. The Reynolds numbers >2300 for blood exiting the dialysis needles suggest blood flow is turbulent in dialysis needles,[26] becoming laminar again within the dialysis access. Anticipated from the models derived above, Figure 2 illustrates that the slope of ΔP changes at the position of the arterial and venous needles, showing a lower slope between the needles as a function of the reduced flow in the access QR between the needles. Of importance, the CFD analysis allows estimation of regional pressure variations induced by needle tip turbulence to provide information about how close a pressure sensor may be to the needle tip while estimating the ΔP along the access between the needles. The flow profiles and needle tip effects were examined using CFD for access flows of 400, 800, and 1200 mL/min with pump on and off at pump rates of 400 mL/min in the center of the lumen and off axis within the dialysis access conduit. We performed CFD analysis under multiple conditions, using pressure tracing as a function of position along the inner diameter of the access and along lines parallel to the axis of the access. These showed constant features as represented in Figure 2, demonstrating that needle tip effects were greatest within 1 cm of the needle tip upstream or downstream from the upstream-facing arterial needle, and within 1 cm upstream of the downstream-facing venous needle, but several centimeters downstream from the venous needle with the dialysis pump on.

### Variable Flow Pressure (VFP) Modeling Results Using Flow Pressure Data

**Figure 3:**
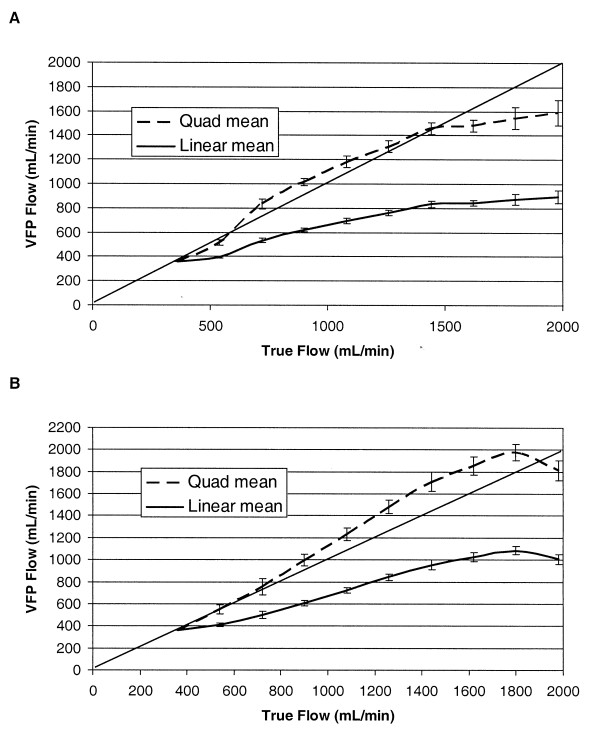
**Variable flow pressure modeling results**. Results of variable flow pressure modeling for (A) 4.76- and (B) 6.35-mm accesses using Equations 4 (linear) and 5 (quadratic), without geometry- or viscosity-dependent terms.

The flow-pressure relationship data were used to test the linear (laminar) and quadratic (turbulent) VFP modeling functions derived above. VFP modeling Equation 4 (linear) and Equation 5 (quadratic) were used to estimate flows, and results are shown in Figures 3A and 3B for 4.76-mm and 6.35-mm-inner-diameter access data, respectively, with standard deviation (10 measurements for each flow) and line of identity shown. It is important to note that these flow estimations used models with no geometry- or viscosity-dependent terms (see derivation of Equations 1 and 3 above).

**Figure 4:**
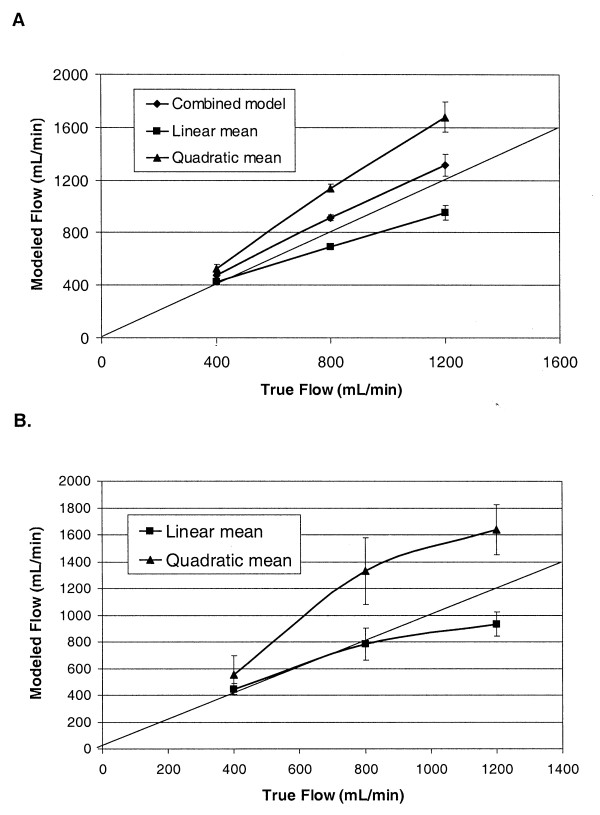
**Experimental flow modeling results**. Experimental flow modeling results for (A) 4.76- and (B) 6.35-mm accesses, without geometry- or viscosity-dependent terms.

As Figure 3 illustrates, VFP modeling Equation 4 (linear model) consistently yielded lower than true volume flow results, and Equation 5 (quadratic model) generally yielded values equal to or above those of true flow. The VFP modeling expressions for linear, quadratic and combined (Equation 8) models were tested using the experimental system in Figure 1 with intraluminal pressure sensing. The results obtained using the experimental system described in the Methods section above are shown for the 4.76- and 6.35-mm-diameter accesses in Figures 4A and 4B, respectively.

Experimental results for the VFP modeling Equation 4 (linear) yielded lower than true volume flow results for the 4.76-mm-diameter access and better approximated the flow in the 6.35-mm-diameter access. The results for Equation 5 (quadratic model) yielded values higher than those of true flow in both access diameters. Results were consistent for sensor needle distances 1, 2, and 5 cm from the dialysis needle tips.

**Figure 5:**
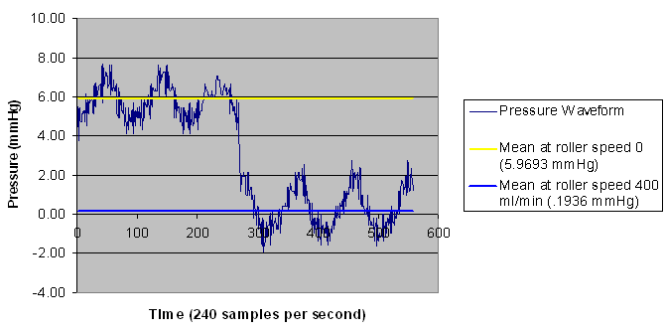
**Real-time waveform results**. Differential pressure waveform of pulsatile flow shifted by turning the pump on. Pulsatile pump flow is 500 mL/min.

Results of real-time waveform information obtained during monitoring are shown in Figure 5. The waveform information reveals that while the pump is off (pump speed = 0), the pulsatility in the pressure gradient between the sensor needles corresponds to the higher pressure gradient and higher flow during systole and correspondingly lower pressure gradients and flows during diastole. When the pump is turned on, an interesting phenomenon is observed: The net pressure gradient between the needles is slightly more than zero. This corresponds to slight net forward flow between the needles while the pump is on. However, what is also seen is that the systolic pressure gradient between the needles is greater than zero during systole, and the diastolic pressure gradient is less than zero. This corresponds to flow in the forward direction during systole and retrograde flow in the access during diastole. Analogous results were seen in a previous study *in vivo*[24] using Doppler measurements of flow between the dialysis needles during dialysis, and the pressure gradients in this experimental system corroborate the prior clinical Doppler flow findings.

The pressure gradients will correspond to alternating flow in either direction and may result in access recirculation depending on the duration of the retrograde flow and needle separation. If the retrograde distance traversed by the blood during the retrograde flow period is greater than the needle separation, then recirculation will develop. The threshold for developing recirculation can be determined by integrating the velocity of reversed (retrograde) blood flow over the time period when flow is reversed within the cardiac cycle. The velocity may be defined simply as v(t) = Q/A, where A is the cross-sectional area and Q is the flow determined from ΔP. A more accurate but complicated Q can be obtained using CFD modeling. For access recirculation to take place, the blood is required to traverse the distance between the needles. This distance D(v, t) for recirculation to develop can be determined by integrating:

(9) $$\text{D}(\text{v},\text{t})=\int_{\text{t}2}^{\text{t}1}\text{v}(\text{t})\text{dt},$$

where t1 is the point in time when retrograde flow starts (when the ΔP signal begins to become negative) during the cardiac cycle, and t2 is the point in time when flow becomes forward again (when the ΔP signal begins to become positive) during the cardiac cycle.

## Discussion

The motivation for investigating these relationships is the desire to have readily available dialysis access flow estimation for use at each treatment, or even multiple times during each treatment, without disrupting the dialysis session. While there is argument about the utility of access flow monitoring, it should be recognized that the current state of flow monitoring technology makes frequent and easy measurements throughout each dialysis treatment impractical. ΔP may allow more frequent monitoring by using either dialysis needle ΔPs or newly evolving MEMS technology for integration of pressure sensors within needle shafts or graft materials.

Since geometric factors must be used for geometry-dependent modeling, ΔP measurements will be based upon approximations or assumptions about graft geometry. As needle separation varies linearly with ΔP, this too will need to be estimated for standard ΔP monitoring strategies. Alternatively, a reference measurement may be made with indicator dilution or Duplex ultrasound to establish a reference flow value when ΔPs are measured. Trends can then be followed at each treatment between periodic reference measurements. Alternatively, in this study, we tested the feasibility of using a geometry-independent flow estimation technique that could be used frequently at each dialysis to improve the accuracy and utility of measurements. Using a combination of quadratic and linear VFP algorithms, true flow may be nearly estimated in grafts on the order of 5- to 6-mm inner diameter typically used in the dialysis setting. Our CFD and experimental results support the possibility of using this method with sensors as close as 1 cm from the dialysis needle tip when the arterial needle faces upstream and the venous needle faces downstream. Alternatively, implantable sensors may be used at greater distances from the dialysis needles. The potential advantages of this or related approaches are based on establishing measurement methods that reduce dependence on access geometry, needle separation distance, and fluid characteristics that may confound other measurement techniques or at least make them more labor intensive to perform.

While ΔP measurements may be obtained from MEMS needle shaft sensors, preliminary data from our laboratory show wider variation in access flow estimation in settings where the access geometry is in the order of 8 mm or larger, such as is encountered with dilated fistulas. Research is ongoing to extend this approach to larger access diameters and more variable access geometries.

In addition, without altering the treatment, diagnostic information may be gathered in real time during dialysis, including continuous pressure waveform monitoring to detect flow reversal that could lead to recirculation. Waveform information has largely been ignored in recent access monitoring literature but may be of additional diagnostic value.[24,27] Parameters derived from waveform information may yield diagnostic information about the compliance and elastic/mechanical properties of the access.

Integrating intraluminal pressure sensors within the dialysis needle may offer advantages in addition to real-time pressure and flow monitoring during dialysis. The location of the sensor could allow real-time detection of the needle migrating out of the lumen prior to the needle tip becoming extra-vascular. Detection of needle migration may decrease the risk of infiltration or bleeding and be a helpful adjunct to monitoring, particularly in settings such as home or nocturnal dialysis. Prior to clinical evaluation, however, the effect of needle tip-induced local flow variances and turbulence, the accuracy and resolution of pressure and placement of the pressure sensors, and the effect of stenosis will all influence the accuracy and practicality of this diagnostic and monitoring approach. These factors will need to be rigorously evaluated in the laboratory and clinical setting.

## Conclusion

In summary, a novel approach to determining access flow from intra-access pressure is presented and the feasibility of determining access volume flow independent of access geometry is examined. While there are clearly multiple factors that must be evaluated such as the effects of access geometries and hemodynamics, variable flow patterns, and the performance of different algorithms, these initial data support further study using differential pressure for dialysis access monitoring.

## Competing Interests

None of the authors have competing interests related to this work.

## Authors' contributions

All authors contributed to the writing of the manuscript. Additionally, WFW performed theoretical background work, designed and conducted experiments, and analyzed data. ZW designed and performed modeling and data analysis. CLC, RB, PP, and HP conducted experimental work and data analysis. And YBG and JMR performed theoretical background work and experimental design.
